# Who Leads, What Matters? Machine Learning and the Complexity of University Performance

**DOI:** 10.1371/journal.pone.0349287

**Published:** 2026-05-28

**Authors:** María Teresa Ballestar, Kathrin Komp-Leukkunen, Jorge Malfeito-Gaviro, Alejandra Ramos, Jorge Sainz

**Affiliations:** 1 Universidad Rey Juan Carlos, Madrid, Spain; 2 Department of Social Sciences, Lappeenranta-Lahti University of Technology (LUT University), Lappeenranta, Finland; 3 IPR; University of Bath, Bath, United Kingdom; Universita degli Studi del Piemonte Orientale Amedeo Avogadro, ITALY

## Abstract

The role of leadership in public institutions, particularly universities, is often linked to goal-setting and decision-making processes that impact efficiency. In Spain, public university rectors are directly elected by academics, staff, and students, offering a unique context for studying leadership influence. This study uses a unique database to analyze Spanish public universities across five categories: academic and research performance, social objectives, internationalization, university characteristics, and rector profiles. Using a K-Means unsupervised machine learning algorithm, we identify five distinct clusters of Spanish public universities, each characterised by a specific combination of institutional performance indicators and management characteristics.

## 1. Introduction

The ability of university managers to impact on the achievement of goals for a higher education institution is a topic that is attracting much interest from academics and policy makers, especially in Europe, where universities face divergent and often changing regulatory frameworks [1]. The diversity of legal frameworks and historical traditions that can be found in continental countries requires a case-by-case analysis of university systems [2]. In this research we focus on the Spanish university system (SUS), marked by a high level of public funding, heterogeneity between universities and inefficiency [3,4].

This study examines the model of university leadership in Spain, characterized by the elective rectorate, a system that diverges significantly from the managerial appointment prevalent in many European contexts [5]. Spanish rectors are elected through a weighted universal suffrage system, a process where each constituent group (tenured professors, contracted faculty and staff, administrative and service personnel, and students) has a proportionate influence on the election outcome based on the number of votes they cast. The specific weighting of these constituent votes is determined by individual university statutes, reflecting a decentralized approach to governance.

The rector, as the institution’s chief executive officer and legal representative, wields substantial authority across a spectrum of domains, including strategic planning and policy implementation, where they are responsible for the articulation and execution of the university’s strategic vision, ensuring alignment with regional and national higher education policies. These responsibilities include the prioritisation of research, teaching, and knowledge transfer initiatives. Fiscal management, encompassing oversight of the university’s budget, including resource allocation to departments and the maintenance of financial sustainability, falls under the rector’s purview.

Human resource management is also the rector’s responsibility, encompassing faculty and staff recruitment, promotion, and evaluation. Furthermore, the rector serves as the university’s primary representative in interactions with external stakeholders, including governmental bodies, funding agencies, industry partners, and other academic institutions. As the university’s legal representative, the rector ensures adherence to all applicable laws and regulations, which is crucial for maintaining the institution’s integrity and reputation.

However, the rector’s authority is subject to some institutional checks and balances. Key limitations include oversight by the University Senate (Claustro Universitario) or Governing Council (Consejo de Gobierno), comprised of representatives from various university sectors. These bodies provide oversight by approving the university’s budget, strategic plan, and other critical policies, ensuring that the rector’s decisions are in line with the university’s overall objectives and the interests of its diverse constituencies.

The result of such a system can lead to a lack of concrete objectives, a lack of rationality in decision-making and policies that are far removed from logic or economic efficiency [6,7]. The immediate consequence is that the definition of Key Performance Indicators (KPIs) proposed by [8] is not necessarily linked to the candidate’s academic and professional prestige, but rather to his or her leadership skills and client networks.

By using a K-Means algorithm, we identify intrinsic patterns and segment data into homogeneous groups. K-Means, being an unsupervised ML method, allows us to explore and discover natural groupings within the data, providing novel and potentially insightful results that might be hidden or inaccessible through traditional econometric methods, such as regression models [9–12].

This research makes several contributions to literature. To the best of our knowledge, we are the first to relate the role of Spanish university rectors and their academic profile in the achievement of universities’ key performance indicators (KPIs) such as academic and research performance, social objectives, internationalization, and other relevant university characteristics. Secondly, methodologically, we use K-Means machine learning methods to characterise the heterogeneity of the Spanish university system. We analyze the impact of each rector in the achievement of basic indicators that can be used as instruments of educational policy. To this end, we have built a database containing information on 28 attributes corresponding to the 47 on-campus public universities distributed in 47 Spanish provinces, 184 campuses and their rectors. These universities manage 9,984 million euros, and in them, just over 103,000 teachers and researchers teach 1.7 million students.

The remainder of the document is structured as follows: Section 2 presents the review of the literature and the theoretical framework; Section 3 describes the database; Section 4 explores the empirical strategies for the database’s analysis; Section 5 discusses the results; and finally, Section 6 presents the conclusions of this research and offers proposals for future research.

## 2. Theoretical framework

Most Western countries recognize, in one form or another, university autonomy in management, especially in academic research. This generates the necessary freedom to foster innovation on the part of professors and should be linked to a target-based funding framework, which leads to an inevitable competition for funds. On the other hand, this competition allows the regulator to create incentives optimizing the provision of teaching and research [13]. Consequently, appropriate processes can be put into place leading to the concentration of research in the most productive academics [14,15].

[16] highlight the importance of personnel management and selection in the economic performance and survival of institutions. In fact, literature has evolved to consider the governance of human resources of any company as a comprehensive system that goes beyond the selection, remuneration and behavior of the organizations, to consider the institution, and how this translates into its performance [17,18].

This premise is not trivial. Mccormack et al. [19] use the method proposed by Bloom & Van Reenen [17] to conclude that the quality of university management has a significant effect on both teaching and research performance (which are not always correlated). From a pool of 248 English universities, the authors use a total of 17 characteristics of university management quality, divided into four groups (operations, supervision, objectives and incentives), assessed through a questionnaire and cross-checked against the Research Excellence Framework (REF), the student satisfaction survey and academic rankings. The results are robust when controlling department size, number of students served, and so on. A first surprising outcome is that the individual characteristics of university leaders do not have a significant effect on the functioning of the university; on the contrary, they do find a positive relationship between the quality of decentralized management, which affects the less strategic roles, such as Human Resources or academic departments, etc.

As opposed to the previous holistic approach, [20,21] focuses on university rectors. She provides empirical foundations through the analysis of a dataset of 400 chancellors and principals from leading American and British business schools. Her hypothesis argues that research-oriented universities experience substantial progress when they are headed by prestigious scholars. She also defends that academics should play leading roles in other organizations, including government bodies responsible for funding educational institutions, as a way of supporting each other. She also finds a positive correlation between research excellence and organizational achievement, proposing that the appropriate level of academic competence in a president will vary according to the university’s research performance and its position in rankings, as well as its future goals. She also develops an empirical model using as a dependent variable the results of the REF of 55 UK universities, conducted in 1992, 1996 and 2001, and as an independent variable cumulative citations over the careers of 157 UK university chancellors in his sample. In short, there is a large literature that theoretically and causally supports the basis for their analysis; good managers generate good results that persist over time [22,23].

The main conceptual limitation in examining managerial ability in higher education is the impossibility of observing it directly. Therein lies an identification problem: we only have results from which to infer the rector’s managerial ability, be it good or bad, and there are no observable yields to estimate the quality of the effect. Following an established line of research on the topic [20,21,24,25], we operationalize managerial ability by academic quality, justified in three arguments. The managerial nature of the rector’s position demands significant domain knowledge on behalf of the top executive, a requirement more readily met, easier to fulfill when the rector is an outstanding researcher [20]. Secondly, the cognitive and behavioural traits that underpin academic excellence are likely to transfer to the skills required for institutional governance [22,26]. In the case of Spanish universities, where the election of the rector is produced by their peers in the academic staff rather than appointed by a governing board, academic prestige is a condition for electoral success and, as such, it has a bearing on institutional power and coalition-building potential. This correlation is imperfect and managerial ability may be unrelated to academic production. Our approach treats the rector quality index as a proxy with measurement error rather than as an assumption of direct causality on managerial ability.

This approach justifies our first question. Hypothesis 1: Do the best-performing universities have the best rectors and the most ambitious goals?

[8] analyze the socio-demographic characteristics of vice-chancellors (and equivalent to vice-chancellors) of British universities, finding a trend towards an increase in social science academics to the detriment of science academics. They also link university objectives with the aspirations of good leaders and all the changes at the top of the university [27]. Their results indicate that, on one hand, newly appointed rectors do not have an immediate effect on the university and that, on the other hand, this effect is not very relevant either and is more linked to the idiosyncrasies of the institution itself. Karadag [26] analyses for Turkey rectors their socio-demographic characteristics, career and academic qualifications measured in terms of scientific output and academic qualifications. His work shows that qualifications have an important impact on the performance of institutions, opening the linkage between research capacity and management capacity to be analyzed in terms of ranking. It also exposes that in a rapidly growing university system, such as the Turkish one, political intervention has a negative effect on the academic performance of universities.

[24] uses the same method as [21] for the universe of Russian universities, but interestingly expands the independent variables by considering some different aspects; while improving the bibliometric variables, classifying universities according to their specialization and differentiating between those rectors who are appointed for their managerial skills and those who are politically selected. His results, which do not contain causality, indicate that being a quality researcher has a long-term effect on institutions, and on productivity in the rector’s own area. In contrast to Karadag [26], the outcome depends on rector’s outstanding academic background and not the procedure followed for his appointment.

This reflection leads us to the second research question of our research. Hypothesis 2: Within the framework of Spanish universities, are the rector’s academic qualifications important for university performance, or does the system of election by referendum override other aspects of university performance?

## 3. Data collection and descriptive analysis.

In this section, we present a descriptive analysis of the original database created specifically for this research. While the following section (Section 4) outlines our analysis, which applies K-Means cluster analysis as the core empirical strategy.

This unique database contains information based on key performance indicators from universities and their leaders identified as relevant in prior literature. It incorporates 27 variables updated up to 2023 for a total of 47 on-campus Spanish public universities. This makes a total of 1,269 datapoints for analysis. The data were collected from various official sources, including the National Institute of Statistics, Scopus, the Spanish Universities Information System, hand-picked data from each university website, and Thomson Reuters SSCI, among others [28].

To correct for the well-documented disciplinary bias of bibliometric indicators, each rector’s h-index and publication count are normalised relative to the mean values of their specific field of knowledge and rescaled to a base-100 index. This procedure ensures that rectors from fields with structurally lower publication volumes—such as humanities or law—are not penalised relative to colleagues from high-output disciplines such as biomedicine or engineering. Comparisons are therefore made exclusively within each scientific field, avoiding spurious cross-disciplinary rankings.

An overview of this database and its variables, their description, characterization and descriptive analysis is provided in Table A1, Appendix A in S1 File. As detailed in Table A1, Appendix A in S1 File, the variables are divided into five categories: Academic and research performance, social objectives, internationalization, university characteristics and rector’s profile. These five categories contain the following information:

i. The academic and research performance category contains information regarding the productivity of the university in terms of number, quality and impact of academic articles published and the ranking of the university according to the SCImago Institutions Ranking.
ii. The social objectives category contains information regarding the accessibility of students to university, scholarships to lower-income students and employability of students 1 and 4 years after finishing their studies.
iii. The internationalization category contains information regarding the presence of international teaching and researching staff at the university and the representativity of international PhD students.
iv. The university category contains information about university budget, size of the university in terms of campuses, students and teaching and researching staff in all the levels of high education.
v. The rector’s characterization category contains information about the rector’s academic performance and additional characteristics such as tenure, the university where the rector earned the doctorate, and the additional synthetic indicator that allows us to rank their professional and academic activity across different areas of knowledge.

## 4. Empirical analysis and methods

In this section we analyse the database using an unsupervised machine learning approach. Specifically, we apply a K-Means segmentation following the CRISP-DM methodology to identify clusters of Spanish public universities with distinct institutional and leadership profiles.

### 4.1. Machine learning methods for segmentation

To answer our research questions, we will use the “*Cross Industry Standard Process for Data Mining*” (CRISP-DM) methodology. While the SEMMA [29]  and the KDD process [30] could be other interesting alternatives, the CRISP-DM process has three advantages. First, the six phases of the CRISP-DM process -Business Understanding, Data Understanding, Data Preparation, Modelling, Evaluation, Deployment- are a natural fit to the structure of the research questions. Second, the CRISP-DM model is the most widely used process model in applied data mining studies, which will foster the replicability of our investigations and their comparison with similar scientific inquiries [31]. Finally, the presence of the Data Understanding and Data Preparation phase in the CRISP-DM methodology perfectly fits in the systematic selection of the six variables provided to the K-Means algorithm out of the initial pool of 28 variables. The variable selection process was therefore based on specific and a priori knowledge and data exploration rather than an arbitrary selection of variables [32].

During the data analysis and data preparation stages, exploration, descriptive and causal analyses were performed, concluding that 9 variables (Table 1) out of 28 candidates (Table 1A, Appendix A in S1 File) were relevant for the development of a ML model based on K-Means segmentation. Most of these variables were also found to be relevant in previous research such as years of seniority of the rector and his or her area of expertise, overlap between the rector’s home university and the university where he or she serves, financial resources available at the university, etc. It is worth noting that one of the selected variables, the percentage of foreign teaching and research staff (perc_TRS_foreign), may partly reflect the rector’s own international academic network rather than a purely institutional characteristic. We treat it as a structural feature of the university, consistent with its role as a contextual variable in the clustering model rather than a direct causal claim about leadership. As we have already pointed out, [25,33] show that the research productivity of departments increases when directors with a notable research profile join them. For this reason, an additional variable is incorporated into the ML model, consisting of a synthetic index that measures the quality of the rector based on four variables on his or her professional activity: years of seniority of the rector in the post, number of publications and h-index, taking into consideration the rector’s area of knowledge. These four rector-related variables—years of seniority, number of publications, h-index, and area of knowledge—are thus aggregated into a single composite indicator (Index_Rector, scaled 0–100), following a field-normalised scoring procedure described in Section 3. This aggregation reduces the initial pool of 9 candidate variables to the 6 variables listed in Table 1, ensuring that the rector’s academic profile is represented by a single, internally consistent measure rather than four correlated sub-indicators.

**Table 1:** Description of the variables used in the Machine Learning (ML) model corresponding to a K-Means segmentation.

Variable	Description	Descriptive Statistics
**SOCIAL OBJECTIVES**		
**perc_student_** **scholarship_holders**	Continuous variable containing the percentage of scholarship students at the respective university.	Mean: 32.51% students Std. Dev: 8.44% students Minimum: 16.60% students Maximum: 47.80% students
**INTERNACIONALIZATION**		
**perc_national_theses**	Continuous variable representing the percentage of domestic doctoral theses at the university.	Mean: 75.92% Std. Dev: 9.73% Minimum: 46.56% Maximum: 94.12%
**UNIVERSITY CHARACTERISTICS**		
**budget2020**	Continuous variable representing the budget managed by each university in 2020.	Mean: 212,441,654€ per university and 61,806,077€ per campus. Std. Dev: 127,512,306€ per university and 46,032,305€ per campus. Minimum: 47,195,312€ per university Maximum: 566,665,273€ per university
**n_TRS**	Discrete variable representing the number of Teaching and Research Staff (TRS) associated with each university.	Mean: 2,200.48 persons Std. Dev: 1,328.23 persons Minimum: 477 persons Maximum: 6,522 persons
**RECTOR’S CHARACTERIZATION**		
**Rector_Dr1_in_house**	Dichotomous variable indicating whether the rector serves at the same university where they earned their doctorate. This variable determines the origin of the rector.	Absolute and relative frequencies: value 0 (NO): 20 rectors (42,55%) value 1 (Yes): 27 rectors (57,44%)
**Index_Rector**	Synthetic indicator of a continuous nature that evaluates from 0 to 100 the quality of the rector based on four metrics of their professional activity: **tenure**, **number of publications, h-Index** and **area of knowledge** (Appendix A. Table A1 in S1 File).	Mean: 62.02% Std. Dev: 22.23% Minimum: 17.02% Maximum: 100%

The definition and descriptive statistics of these six variables included in the ML model are detailed in Table 1.

The chosen segmentation method is K-Means, an unsupervised ML method that is widely adopted for segmentation in academia and business [34]. It is an unsupervised method, as it does not need a target variable to perform the classification, although it does require the establishment of the number of segments (clusters) a priori. Instead of trying to predict a response variable, it performs a classification of the input data by identifying patterns [35]. This results in segments of observations that are highly homogeneous within each group, yet highly heterogeneous between groups. The main advantage over other segmentation methods is that K-means work accurately for samples as small as 50, as is our [36–38], while the main disadvantage is the need to establish the number of segments K a priori [39], nevertheless this inconvenience can be avoided by using genetic algorithms for clustering. Genetic algorithms use a random number (k) of clusters, not defined by the user, ranking between 2 to *√n* [40].

**Fig 1:**
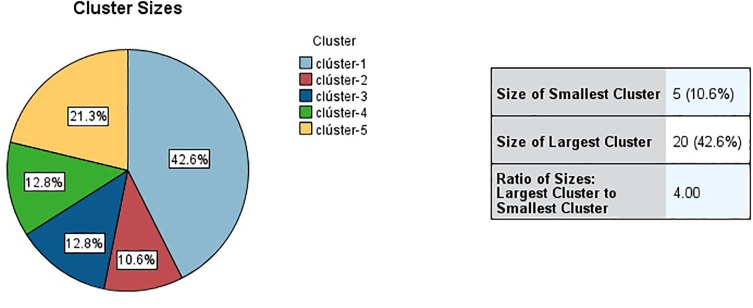
Distribution of Spanish universities in clusters. Source: authors’ own elaboration.

Our model classifies public universities into 5 distinct clusters, based on their characteristics and those of their rectors. The percentage of universities in each group is Cluster 1: 42.6%; Cluster 2: 10.6%; Cluster 3: 12.8%; Cluster 4: 12.8%; Cluster 5: 21.3% (Fig 1). The taxonomy of the clusters is presented in Table 2. The fit of the K-Means model is measured with the Silhouette coefficient, which captures the consistency of the structure of the segments, examining the degree of homogeneity in each segment and the degree of separation between them. Its minimum value is −1 and the maximum value is 1, and robust models have values for this coefficient between 0.5 and 1 [41–43]. In this model the value of the Silhouette coefficient is 0.513, making it robust [44]. The importance of the variables measures how relevant they are in the model, with the minimum value being 0 and the maximum value being 1.

**Table 2:** Cluster taxonomy: Centroids for continuous variables and frequencies for dichotomous variables.

		CENTROIDS									
		perc_student_ scholarship_holders		N_TRS		index_Rector		Perc_national_ theses		budget2020	
**Cluster (Frequencies)**	**Number of Universities**	**Mean**	**Standard Deviation**	**Mean**	**Standard Deviation**	**Mean**	**Standard Deviation**	**Mean**	**Standard Deviation**	**Mean**	**Standard Deviation**
**Cluster 1**	20	33.25	7.75	1.43K	0.69K	64.06	19.72	78.08	10.34	133,190K€	72,245K€
**Cluster 2**	5	29.26	6.72	5.14K	1.00K	63.45	15.66	79.30	4.33	460,602K€	76,290K€
**Cluster 3**	6	22.95	5.92	2.91K	0.75K	65.54	28.08	65.63	11.89	309,115K€	53,323K€
**Cluster 4**	6	42.22	5.63	2.17K	0.79K	31.10	11.47	76.19	6.39	227,258K€	124,902K€
**Cluster 5**	10	36.40	6.01	1.86K	0.57K	73.69	16.68	79.60	7.06	179,972K€	59,439K€
**Combined**	**47**	**33.33**	**8.44**	**2.20K**	**1.33K**	**62.03**	**22.24**	**76.70**	**9.73**	**212,442K€**	**127,512K€**
		**FREQUENCIES**									
		**Rector_Dr1_in_house**									
		**Frequencies**		**Percentages**							
**Cluster (Percentages)**	**Number of Universities**	**0 (No)**	**1 (Yes)**	**0 (No)**	**1 (Yes)**						
**Cluster 1**	20	20	0	100%	0%						
**Cluster 2**	5	0	5	0%	19%						
**Cluster 3**	6	0	6	0%	22%						
**Cluster 4**	6	0	6	0%	22%						
**Cluster 5**	10	0	10	0%	37%						
**Combined**	**47**	**20**	**27**	**100%**	**100%**						

Additionally, alongside the Silhouette coefficient, five one-way ANOVA tests and one Chi-square test were conducted to confirm these significant differences between the clusters in terms of the six variables used in the K-Means ML model. The goal of the ANOVA and Chi-square testing was to confirm the suitability of these variables for clustering [45,46].

The five one-way ANOVA tests applied on the quantitative variables revealed significant differences among clusters in terms of percentage of scholarship students (“perc_students_scholarship_holders”), number of Teaching and Research Staff (TRS) (“n_TRS”) and budget managed by each university in 2020 (“budget2020”), with p-values less than 0.001. The synthetic indicator, which evaluates the quality of the rector on a scale from 0 to 100 (“Index_Rector”), had a p-value of 0.002. Finally, the p-value for percentage of domestic doctoral theses at each university (“perc_national_theses”) was 0.043. The Chi-square test applied on the dichotomic variable indicating whether the rector serves at the same university (“Rector_Dr1_in_house”) where they earned their doctorate showed that there are also significant differences between the clusters with p-value 0.001. These findings support the robustness of this segmentation.

The variable importance scores reported refer to the relative contribution of each variable to cluster separation in the K-Means model and should not be confused with classical statistical significance (i.e., p-values from hypothesis tests). These scores are derived from the ratio of between-cluster variance to total variance for each variable—analogous to an eta-squared measure—and are bounded between 0 and 1, where 1 indicates that a variable is perfectly discriminating across clusters (Ballestar et al., 2018). The ANOVA p-values reported in the preceding paragraph confirm that all six variables exhibit statistically significant differences across clusters; the importance scores here provide a complementary ranking of their discriminatory power. The two variables with the highest cluster-separation scores are number of Teaching and Research Staff (“n_TRS”) and budget in 2020 (“Budget2020”), with scores of 1.00 and 0.83 respectively, reflecting that university size is the dominant axis of differentiation. These are followed at a considerable distance by the percentage of scholarship holders (“perc_student_scholarship_holders”, score 0.34), the rector’s quality index (“Index_Rector”, score 0.24), and the percentage of national doctoral theses (“perc_national_theses”, score 0.13). The variable with the lowest discriminatory power is the binary indicator of the rector’s academic origin (“Rector_Dr1_in_house”), which contributes positively to the overall cluster structure.

## 5. Results and discussion

This section explains the characteristics of each cluster of universities and their respective rectors.

a) ***External origin of the rector and small university size.***

The origin of the rector, understood as if he/she is rector of the university where he/she got his/her PhD, as the legal representative and first executive authority of the university is very relevant when segmenting Spanish universities. These findings are descriptively consistent with the pattern underlying Hypothesis 2: the historical relationship between the rector and the university, combined with the system of election by referendum, appears to be associated with institutional clustering patterns that go beyond the rector’s academic performance. However, given the descriptive nature of cluster analysis, causal claims cannot be established on this basis alone.

**Cluster 1** (Fig 1; Table 2) is the largest, accumulating 42.55% of the universities (20 universities) and has a rector who holds a position at a university where he/she did not do a doctorate. The rector’s quality index is slightly above the average (+3.28%) with 64.06 points. It is worth noting that 45% of these rectors belong to the scientific-technical area of knowledge (25% engineers and 20% biologists).

The most notable characteristic of these universities is that they are the smallest, and therefore also have the smallest budgets: they have an average of 3 campuses, 17,000 students and managed a budget of €133.19M in 2020, −23.37%, −30.52% and −37.31% lower than the national average respectively.

This finding shows that there is homogeneity between universities managed by rectors who did not have a doctorate from the university, as well as the preference of larger universities to have executive leaders who have developed within the university itself.

b) ***Large universities.***

**Cluster 2** (Fig 1; Table 2) accumulates 10.64% of the universities (5 universities). These have in common that they are large and that they are managed by rectors coming from areas of knowledge such as economics, psychology, chemistry and veterinary science at these universities. The rector’s index is slightly above average (+2.30%) with 63.45 points.

They have an average of 5.6 campuses, with 54,000 students and managed €460.60M of budget in 2020, + 43.04%, + 120.29% and +116.81% higher than the national average respectively.

On the other hand, although the number of students is higher, the percentage of grant holders is notably lower than the national average (−12.20%). Furthermore, in the field of research, they are committed to national doctoral theses, with a percentage of 79.30% (+3.38% vs. the national average).

c) ***Large universities that are committed to international research.***

**Cluster 3** (Fig 1; Table 2) accounts for 12.77% of the universities (6 universities). What these universities have in common is that they are large universities and are committed to international research, but at the same time, they have a much lower number of scholarship holders than the national average. They are managed by rectors from scientific-technical knowledge areas (physics, engineering and chemistry) of these universities and their quality index is considerably above the average (+5.67%) with 65.54 points.

These universities have an average of 4.83 campuses, with 31,000 students and managed €309.11M of budget in 2020, + 23.46%, 28.02%, + 45.51% higher than the national average respectively.

It is particularly noteworthy that, although the number of students is higher than average, these universities have a given number of scholarship holders well below the national average (−31.13%), which contrasts with their commitment to internationalization in research, reaching 34.37% of international doctoral theses (−14.43% fewer national theses than average).

d) ***Universities with rectors with low quality index and high percentage of scholarship holders.***

**Cluster 4** (Fig 1; Table 2) accumulates 12.77% of the universities (6 universities). These have in common that they are slightly above average in size, they are large recipients of scholarship students, and their respective rectors have a quality index well below the average (−49.87%) with 31.10 points. Fifty percent of these rectors come from law fields and fifty percent from engineering, medicine and chemistry fields of the same universities where they studied.

These universities have an average of 4.33 campuses, with 26,000 students and managed €227.25M of budget in 2020, + 10.69%, + 6.26%, + 6.97% higher than the national average respectively.

It is worth noting that these universities are characterized by the fact that they host students on scholarships (42.22% are on scholarships, + 26.68% higher than the national average). On the other hand, it is the only group of universities that presents an imbalance between the ratio of teaching and research staff and the number of students. In this group of universities, the number of students is + 6.26% higher than the national average, while the number of teaching and research staff is −1.31% below the national average.

e) ***Universities with rectors with a high-quality index.***

**Cluster 5** (Fig 1; Table 2) is the second largest cluster, accumulating 21.28% of the universities (10 universities) and has a rector who works at the same university where he/she did the doctorate. What these universities have in common is that their size is below average and that their respective rectors have a quality index that is well above average (+18.81%) with 73.69 points. Forty percent of these rectors come from law, and the rest from other fields such as biology, economics, physics, history, medicine and chemistry.

These universities have an average of 4.10 campuses, with 19,000 students and managed €179.97M of budget in 2020, 4.73% higher, −19.68% and −15.28% lower than the national average.

The quality index of their rectors, who lead universities that also host a significant percentage of scholarship holders, + 9.23% higher than the national average, and who are committed to theses of national origin, reaching the highest national percentage with 79.60% (+3.78%), stands out very notably.

## 6. Conclusions

This paper offers a novel approach to analyzing university leadership by applying K-Means cluster analysis to a comprehensive database of Spanish public universities. Rather than assuming a linear causal relationship between rector characteristics and institutional outcomes, the clustering approach reveals the natural typology of universities and the profiles of their leaders, providing a descriptively rich and methodologically robust account of institutional heterogeneity in the Spanish higher education system.

By focusing on the Spanish university system, characterized by its elective rectorate model, the research offers insights into a governance structure that differs significantly from the managerial appointment prevalent in many European contexts. The multidimensional performance assessment, which considers a wide range of key performance indicators (KPIs) in academic and research performance, social goals, internationalization, and university characteristics, provides a holistic view of university performance. In terms of practical implications, the findings can guide educational policy by providing empirical evidence on the impact of leadership profiles on university performance, which can inform decisions on governance structures and appointment processes for university leaders.

The research also highlights the importance of academic credentials and management skills for effective university leadership, which can inform professional development programs for current and aspiring university leaders. The study provides information that can help university managers optimize resource allocation and strategic planning by identifying patterns and relationships between leadership characteristics and various performance indicators. The synthetic index created to measure rector quality across different knowledge domains provides a standardized method for comparing leadership effectiveness, which can be valuable for institutional assessment and improvement.

The study’s findings on the elective rector system in Spain can inform debates on the effectiveness of different university governance models, potentially influencing reform efforts in higher education systems worldwide. By addressing these theoretical and practical issues, the article contributes significantly to understanding university leadership and its impact on institutional performance, providing a basis for future research in this area and offering practical information for improving higher education governance and management.

Specifically, our results show that only smaller universities in Cluster 1 have opted to recruit external talent for their management. This finding shows that there is great homogeneity among the universities managed by these rectors. Large universities (Clusters 2 and 3), which receive the greatest volume of students and resources, are also those with the lowest percentage of scholarship students. Universities in Cluster 4, with rectors with quality indices well below the national average are the ones that lead universities with the highest percentage of scholarship students. This group of universities also shows an imbalance between the ratio of teaching and research staff and the number of students, which could indicate insufficient resources compared to the rest of the universities that do not have so many students in need of grants. Finally, the rectors with the highest quality index, located in universities below the average size (Cluster 5), also host a large percentage of scholarship holders in their universities and become local research promoters with the highest percentage of national theses. Taken together, these findings provide an answer to the two hypotheses. Hypothesis 1 is only partially supported: the cluster with the highest rector quality index (Cluster 5) is associated with below-average institutional size, indicating that academic merit alone does not translate into superior overall performance. University size is the dominant structural axis differentiating clusters, not rector quality. Regarding Hypothesis 2, the cluster analysis reveals that the most discriminating categorical variable is whether the rector completed their doctorate at the same institution (Rector_Dr1_in_house), which perfectly separates Cluster 1 from all others. This descriptive pattern is consistent with the notion that the elective system rewards institutional embeddedness over academic merit, particularly in smaller universities. However, cluster analysis does not establish causality; formal econometric analysis would be required to test this hypothesis rigorously.

Our research has several shortcomings. The first, and perhaps the most important, is the static nature of the database. The analysis gathers the latest data available at the time, but it does not include the dynamics of the changes of rectors and how the change of leadership affects the university’s characteristics and performance, for which it would be necessary to build a panel of these dynamics. The lack of collaboration on the part of the official institutions has prevented us from expanding the sample. It would also be possible to broaden the sample to include the organizational characteristics of universities and the management capacity of their leaders, as [7,8,27] does. Finally, in the spring of 2023 a new university law was passed in Spain which, although it does not change the election system, has modified some of the organizational responsibilities of the rectors, which opens a new dynamic of analysis for the future.

## Supporting information

S1 File Appendix A Table A1: Database Description. (DOCX)
