# Supplementary material for: Who Leads, What Matters? Machine Learning and the Complexity of University Performance
Source: PLoS One. 2026 May 28;21(5):e0349287. doi: 10.1371/journal.pone.0349287 (PMC13218531; doi:10.1371/journal.pone.0349287)
Supplement: S1 File — (DOCX) [file pone.0349287.s001.docx]

**Appendix A. Table A1:** Database Description

| **Variable** | **Description** | **Descriptive Statistics** |
| --- | --- | --- |
| **ACADEMIC PERFORMANCE** | | |
| **exaly_articles** | Discrete variable which contains the number of academic articles published by the university according to Exaly (Open data base of authors and journals). | Mean: 18,374.19 articles Std. Dev: 16,364.50 articles Minimum: 897 articles Maximum: 80,700 articles |
| **exaly_citations** | Discrete variable which contains the number of citations of academic articles published by the university according to Exaly (Open data base of authors and journals). | Mean: 426,291.48 citations Std. Dev: 454,541.56 citations Minimum: 16,700 citations Maximum: 2,500,000 citations |
| **exaly_h_index** | Discrete variable which contains the h-Index from the academic articles published by the university according to Exaly (Open data base of authors and journals). | Mean: 177.55 Std. Dev: 65.30 Minimum: 88 Maximum: 413 |
| **Scimago_research_Spain** | Discrete variable representing the research ranking of Spanish universities, according to the SCImago Institutions Ranking. | Mean: 24.55 Std. Dev: 14.36 Minimum: 1 Maximum: 49 |
| **Scimago_overall_Spain** | Discrete variable representing the overall ranking of Spanish universities, according to the SCImago Institutions Ranking. | Mean: 30.91 Std. Dev: 39.40 Minimum: 1 Maximum: 274 |
| **Scimago_research_global** | Discrete variable representing the research ranking of Spanish universities worldwide, according to the SCImago Institutions Ranking. | Mean: 1,412.94 Std. Dev: 652.55 Minimum: 240 Maximum: 2,763 |
| **Scimago_overall_global** | Discrete variable representing the overall ranking of Spanish universities worldwide, according to the SCImago Institutions Ranking. | Mean: 1,489.79 Std. Dev: 1,067.27 Minimum: 94 Maximum: 4,040 |
| **SOCIAL OBJECTIVES** | | |
| **perc_student_**  **scholarship_holders** | Continuous variable containing the percentage of scholarship students at the respective university. | Mean: 32.51% students Std. Dev: 8.44% students Minimum: 16.60% students Maximum: 47.80% students |
| **avg_duration_degree** | Continuous variable representing the average number of years students take to complete a degree at the university. | Mean: 4.83 years Std. Dev: 0.32 years Minimum: 4.43 years Maximum: 6.08 years |
| **perc_affiliation_1year** | Continuous variable representing the percentage of students who secure employment one year after completing their degrees at university. | Mean: 47.10% Std. Dev: 9.12% Minimum: 30.51% Maximum: 67.37% |
| **perc_affiliation_4years** | Continuous variable representing the percentage of students who secure employment four years after completing their degrees at university. | Mean: 73.29% Std. Dev: 5.08% Minimum: 60.86% Maximum: 84.40% |
| **avg_access_grades** | Continuous variable representing the average entry grades required for degree programs at the university. | Mean: 9.66 points Std. Dev: 0.67 points Minimum: 8.13 points Maximum: 12.00 points |
| **INTERNACIONALIZATION** | | |
| **perc_TRS_foreign** | Continuous variable representing the percentage of Teaching and Research Staff (TRS) originating from countries other than Spain. | Mean: 2.49%  Std. Dev: 2.04% Minimum: 0.72% Maximum: 12.51% |
| **perc_national_theses** | Continuous variable representing the percentage of domestic doctoral theses at the university. | Mean: 75.92% Std. Dev: 9.73% Minimum: 46.56% Maximum: 94.12% |
| **UNIVERSITY CHARACTERISTICS** | | |
| **budget2020** | Continuous variable representing the budget managed by each university in 2020. | Mean: 212,441,654€ per university and 61,806,077€ per campus. Std. Dev: 127,512,306€ per university and 46,032,305€ per campus. Minimum: 47,195,312€ per university Maximum: 566,665,273€ per university |
| **budget_per_student2020** | Continuous variable representing the budget per student managed by each university in 2020. | Mean: 8,690.87€ per student Std. Dev: 1,470.76€ per student Minimum: 4,207.10€ per student Maximum: 11,848.70€ per student |
| **n_degree_students** | Discrete variable representing the number of graduate students at the university. | Mean: 20,202.68 students Std. Dev: 11,984.96 students Minimum: 3,582 students Maximum: 52,757 students |
| **n_master_students** | Discrete variable representing the number of master's students at the university. | Mean: 2,731.40 students Std. Dev: 1,727.67 students Minimum: 573 students Maximum: 7,068 students |
| **n_doctoral_students** | Discrete variable representing the number of doctoral students at the university. | Mean: 1,800.59 students Std. Dev: 1,344.54 students Minimum: 303 students Maximum: 6,319 students |
| **n_campuses** | Discrete variable representing the number of campuses associated with each university. | Mean: 3.91 campuses Std. Dev: 1.77 campuses Minimum: 1 campus Maximum: 8 campuses |
| **n_TRS** | Discrete variable representing the number of Teaching and Research Staff (TRS) associated with each university. | Mean: 2,200.48 persons Std. Dev: 1,328.23 persons Minimum: 477 persons Maximum: 6,522 persons |
| **n_doctoral_programmes** | Discrete variable representing the number of doctoral programmes available at each university. | Mean: 28.57 programmes Std. Dev: 16.90 programmes Minimum: 4 programmes Maximum: 71 programmes |
| **RECTOR'S CHARACTERIZATION** | | |
| ***Rector's Academic Performance*** | | |
| **Rector_h-index** | Continuous variable representing the rector's h-index. | Mean: 21.25 Std. Dev: 16.63 Minimum: 4 Maximum: 93 |
| **Rector_area** | Categorical variable representing the rector's area of academic specialization. | Absolute and relative frequencies: Engineering: 9 (19.14%) Law: 8 (17.02%) Chemistry: 6 (12.76%) Biology: 5 (10.63%) Medicine: 4 (8.51%) Economy: 3 (6.38%) Physics: 3 (6.38%) Veterinary: 3 (6.38%) Philosophy: 2 (4.25%) Psychology: 2 (4.25%) History: 1 (2.12%) Mathematics: 1 (2.12%) |
| ***Other Rector's characteristics*** | | |
| **Rector_Dr1_in_house** | Dichotomous variable indicating whether the rector serves at the same university where they earned their doctorate. This variable determines the origin of the rector. | Absolute and relative frequencies: value = 0 (NO): 20 rectors (42,55%) value = 1 (Yes): 27 rectors (57,44%) |
| **Rector_tenure** | Discrete variable indicating the number of years the rector has been in their position at university. | Mean: 3.72 years Std. Dev: 2.41 years Minimum: 0 years Maximum: 8 years |
| **Rector_publications** | Discrete variable indicating the number of academic publications by the rector. | Mean: 110.57 publications Std. Dev: 113.08 publications Minimum: 22 publications Maximum: 728 publications |
| **Index_Rector** | Synthetic indicator of a continuous nature that evaluates from 0 to 100 the quality of the rector based on four metrics of their professional activity: tenure, number of publications, h-Index and area of knowledge. | Mean: 62.02% Std. Dev: 22.23% Minimum: 17.02% Maximum: 100% |
